# Applying the ARRIVE Guidelines to an In Vivo Database

**DOI:** 10.1371/journal.pbio.1002151

**Published:** 2015-05-20

**Authors:** Natasha A. Karp, Terry F. Meehan, Hugh Morgan, Jeremy C. Mason, Andrew Blake, Natalja Kurbatova, Damian Smedley, Julius Jacobsen, Richard F. Mott, Vivek Iyer, Peter Matthews, David G. Melvin, Sara Wells, Ann M. Flenniken, Hiroshi Masuya, Shigeharu Wakana, Jacqueline K. White, K. C. Kent Lloyd, Corey L. Reynolds, Richard Paylor, David B. West, Karen L. Svenson, Elissa J. Chesler, Martin Hrabě de Angelis, Glauco P. Tocchini-Valentini, Tania Sorg, Yann Herault, Helen Parkinson, Ann-Marie Mallon, Steve D. M. Brown

**Affiliations:** 1 Mouse Informatics Group, Wellcome Trust Sanger Institute, Cambridge, United Kingdom; 2 Samples, Phenotypes and Ontologies, European Molecular Biology Laboratory—European Bioinformatics Institute, Cambridge, United Kingdom; 3 Mammalian Genetics Unit, Medical Research Council, Harwell, United Kingdom; 4 The Wellcome Trust Centre for Human Genetics, Oxford University, Oxford, United Kingdom; 5 Cellular Genetics, Wellcome Trust Sanger Institute, Cambridge, United Kingdom; 6 Toronto Centre for Phenogenomics, Mount Sinai Hospital, Toronto, Canada; 7 BioResource Center, RIKEN, Tsukuba, Japan; 8 Mouse Genetics Project, Wellcome Trust Sanger Institute, Cambridge, United Kingdom; 9 Mouse Biology Program, University of California Davis, Davis, California, United States of America; 10 Molecular Physiology and Biophysics, Baylor College of Medicine, Houston, Texas, United States of America; 11 Molecular and Human Genetics, Baylor College of Medicine, Houston, Texas, United States of America; 12 Genetics, Children's Hospital Oakland Research Institute, Oakland, California, United States of America; 13 Genetics, The Jackson Laboratory, Bar Habour, Maine, United States of America; 14 German Mouse Clinic-Institute of Experimental Genetics, Helmholtz Zentrum München, Neuherberg, Germany; 15 Experimental Genetics—Center of Life and Food, Technische Universität München, Freising-Weihenstephan, Germany; 16 Monterotondo Mouse Clinic, CNR Institute of Cell Biology and Neurobiology, Monterotondo, Scalo, Italy; 17 PHENOMIN, Centre National de la Recherche Scientifique, Illkirch, France; 18 Institut Clinique de la Souris, Université de Strasbourg, Illkirch, France; 19 Institut de Génétique et de Biologie Moléculaire et Cellulaire, Institut National de la Santé et de la Recherche Médicale U964, Illkirch, France

## Abstract

The Animal Research: Reporting of In Vivo Experiments (ARRIVE) guidelines were developed to address the lack of reproducibility in biomedical animal studies and improve the communication of research findings. While intended to guide the preparation of peer-reviewed manuscripts, the principles of transparent reporting are also fundamental for in vivo databases. Here, we describe the benefits and challenges of applying the guidelines for the International Mouse Phenotyping Consortium (IMPC), whose goal is to produce and phenotype 20,000 knockout mouse strains in a reproducible manner across ten research centres. In addition to ensuring the transparency and reproducibility of the IMPC, the solutions to the challenges of applying the ARRIVE guidelines in the context of IMPC will provide a resource to help guide similar initiatives in the future.

## Abstract 2

Transparent reporting is key to ensuring reproducibility of animal research. This article examines the challenges of applying the ARRIVE guidelines to a large-scale, collaborative, in vivo research initiative—the International Mouse Phenotyping Consortium.

## Introduction

**Fig 1:**
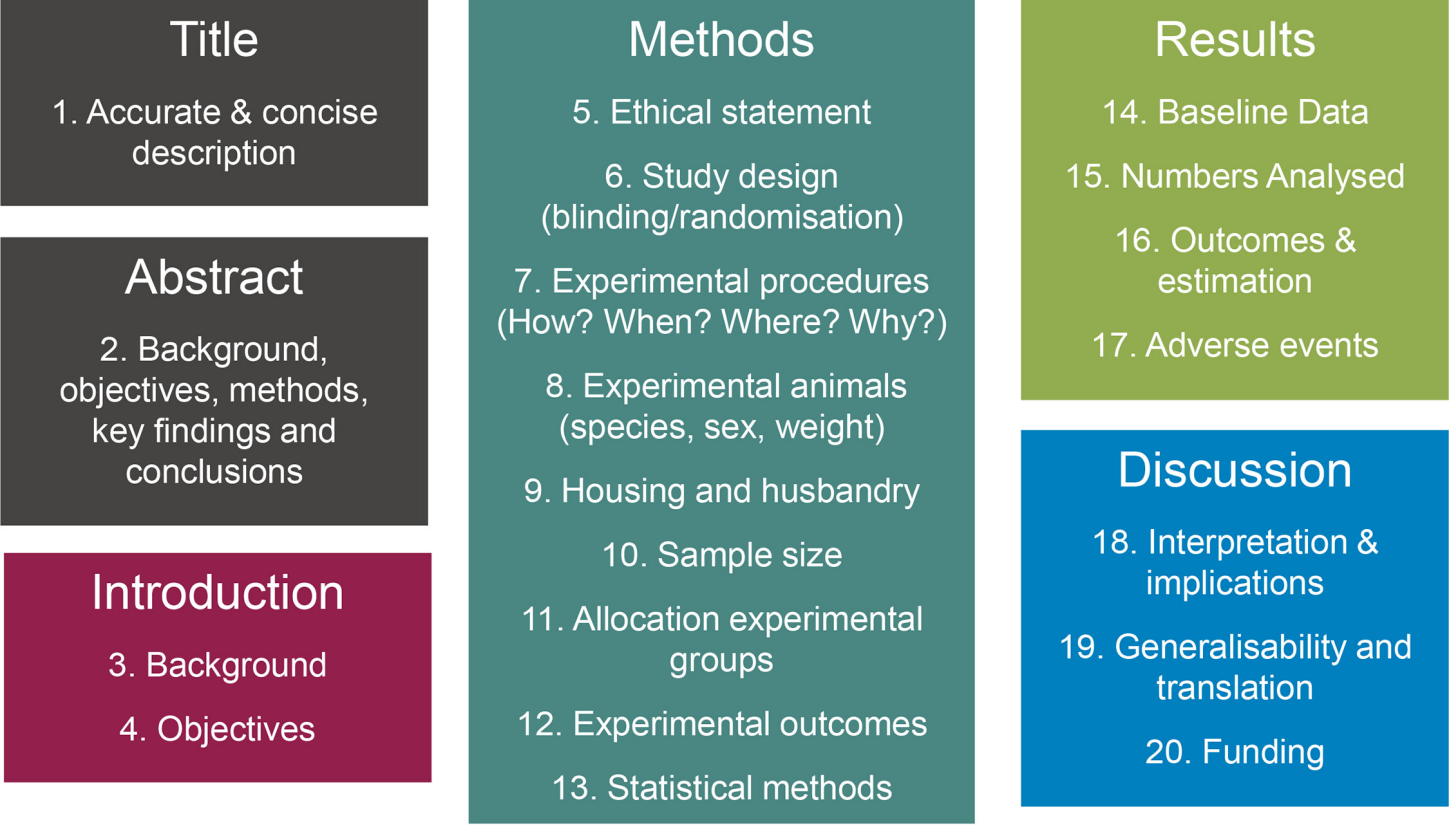
A summary of the areas encompassed by the ARRIVE guidelines. The ARRIVE guidelines are organised into twenty sections, which cover all areas of a typical research manuscript. *Image credit*: NC3Rs: https://www.nc3rs.org.uk/arrive-guidelines.

In 2010, the Animal Research: Reporting of In Vivo Experiments (ARRIVE) guidelines were published [1] to address the growing concerns with poor experimental design and lack of transparent reporting of in vivo experiments in the published literature [2–4]. The guidelines consist of a checklist of 20 items, which are considered key to describing a study comprehensively and transparently (Fig 1). Transparent reporting is a fundamental component required to ensure reproducibility of animal research and allow later inclusion in meta-analytic studies [5]. To our knowledge, no specific guidelines exist for an in vivo database, and there has been no report of the use of the ARRIVE guidelines to inform the collection, analysis, and presentation of data in a pre-publication database. Yet the transparency needed is equally essential in this situation.

The International Mouse Phenotyping Consortium (IMPC) [6] aims to phenotype knockouts for all protein coding genes in the mouse genome and hence provide an invaluable resource to the community, given that the mouse is the premier model organism for understanding gene function in development and disease. The IMPC community has defined a core set of procedures to assess multiple biological systems and has developed a central resource (www.mousephenotype.org) to disseminate the data. The database contains continuous, categorical, count, and image data for over 734,284 experiments on 46,972 mice as of January 2015.

Here, we describe how the IMPC applied the ARRIVE guidelines to a large data resource and highlight the challenges and how we addressed them. Applying the ARRIVE guidelines has required extensive development, which has not been confined to the database and web portal but has also impacted on communication and understanding within the community. Achieving transparency is not a simple task but has benefits not only to the consumers of the data but also the experimenters and is critical for the value of the research to be fully realised.

### The Aims and Challenges of Setting Up a Large-Scale Database

A large-scale database aims to consolidate information into one site for maximum exposure and to add functionality to mine and explore data. The scale of the project, with many types of variables and data from many sources, brings many challenges in capturing the data (defining what to capture and methods of capture), data organisation, and data presentation. A significant challenge for a database portal is how to organise the information in a logical way that leads to readable pages that do not overwhelm a user upon first inspection. The ARRIVE guidelines were developed specifically for peer-reviewed scientific publications of in vivo data, but the goals of transparency are equally applicable to databases and critical to allow reproducible research. We therefore decided to use the ARRIVE guidelines to review and subsequently refine our approach to maximise the value of the data we were presenting.

## Application of the ARRIVE Guidelines

### ARRIVE Guidelines—Introduction—Background and Objective (Items 3 and 4)

The IMPC home page, http://www.mousephenotype.org/goals-and-background, details the goals of the project and the relevance to human biology.

### ARRIVE Guidelines—Methods—Ethical Statement (Item 5) and Funding Information (Item 20)

IMPC includes ten institutes collecting phenotyping data, guided by their own ethical review panels, licenses, and accrediting bodies that reflect the national and/or geo-political constructs in which they operate. The ethical and funding information were not initially captured, but our review steered by the guidelines drove us to develop an ethical and funding survey. The survey provides a transparent view and enables prospective users of the data to understand the ethical framework in which the data were collected.

### ARRIVE Guidelines—Methods—Experimental Procedures (Item 7) and Experimental Outcomes (Item 12)

The experimental procedures in IMPC are defined by standardised data format in the International Mouse Phenotyping Resource of Standardised Screens (IMPReSS) database (https://www.mousephenotype.org/impress/). IMPReSS is based on a pipeline concept, which is a series of experimental procedures performed in a specified order. Each procedure defines the purpose, experimental design, the protocol, the measured variables, potential QC failure criteria, methodology, variables measured, and the metadata to be captured. The information is organised such that each data point can be associated with the relevant procedural and mouse information.

To provide traceability, IMPReSS also stores a record of change histories. IMPReSS thus provides the framework not only for transparency in the procedure and what data are required to be captured but also stores information to facilitate subsequent analysis, hence making it the backbone of the database and web portal. The development of this resource has required extensive collaboration with the institutes, area experts, and database engineers.

### ARRIVE Guidelines—Methods—Experimental Animals (Item 8)

The scale of the project necessitated the development of an International MicroInjection Tracking System (iMITS), which is the repository of the genotypes and coordinates production and phenotyping. The scale has also required an automated method of data capture from phenotyping centres. It is supported by a strict data standard that defines, in addition to the variables defined in the procedures at IMPReSS, data about each animal (Local ID, date of birth, strain, sex, and centre).

### ARRIVE Guidelines—Methods—Housing and Husbandry (Item 9) and Results—Adverse Events (Item 17)

To capture housing and husbandry information, the community came together and, based on the ARRIVE guidelines, the Gold Standard Publication Checklist reporting guidelines [7], and the Genetically Altered (GA) Passport [8], constructed a series of questions and answers that was used to develop a housing and husbandry survey. The phenotyping pipelines have been designed to ensure that there are no welfare-related issues for a normal mouse; however, incidental welfare issues may arise from the environment and the genetic background of the mice. Animals presenting with these incidental welfare issues will be assessed on a case-by-case basis to determine if the issue can be managed through remedial care, such as wet mash on the cage floor for runting or clipping teeth in the case of malocclusions, or whether our ethical obligation to the animals and the scientific endpoints are better served by euthanizing the affected animal and providing a replacement. In addition to these incidental welfare concerns, a welfare issue could occur as a consequence of the genetic alteration carried by the mice. Two complementary approaches are used to identify and manage these welfare issues. First, during the generation of the first homozygous progeny, we assess basic dysmorphology. If significant welfare concerns are raised, then the breeding strategy is modified to discontinue the generation of homozygous mice and heterozygous mice are phenotyped instead. In addition to this early assessment, ongoing monitoring is carried out through the lifetime of the colony. When animals do present a welfare concern, either an intervention is made in the form of the Alternate Mouse Pipeline or, when applicable, remedial action is taken within husbandry (e.g., long water spouts or food on cage base) or within experimental procedures (e.g., an alternate anesthetic is used or selected tests are omitted). The Alternate Mouse Pipeline is a bespoke phenotyping pipeline in which the age and procedures are adapted to manage the welfare of the line. Currently the community is in the process of defining the Alternate Mouse Pipeline within IMPReSS that will, in the future, allow this data to be captured and disseminated. These two complementary approaches allow us to maximise the value of information extracted from the lines while minimising welfare issues. If mice or cryopreserved sperm are requested, any welfare concerns are provided in a GA passport [8].

### ARRIVE Guidelines—Methods—Study Design (Item 6) and Allocating Animals to Experimental Groups (Item 11)

As a high-throughput project, the community is following a general design of testing seven animals per sex for each knockout strain to be compared to the control data. Within the analysis we consider the mouse as the experimental unit. Since we cannot randomly allocate animals to experimental groups, we rely on Mendelian inheritance to provide the randomisation method. However, there are still many other aspects of the experiment where planning is necessary to avoid bias (e.g., order effects). Whilst the general approach and procedures are captured and tightly defined in IMPReSS, implementation (e.g., blinding) could vary significantly between institutes. Discussions on study design and implementation identified terminology and usage differences as a significant impediment to communication within the consortium. We therefore developed a standardised language to describe various aspects of the experimental design. The resulting Mouse Experimental Design Ontology (MEDO) is hosted at the BioPortal for biomedical ontologies (http://bioportal.bioontology.org/ontologies/MEDO) For example, the phrase “control mouse” in the context of a gene knockout was initially thought to have no ambiguity. In fact, there were four different possible control designs that could be implemented (Table 1). A similar issue affects potential confounders since a strategy followed by one institute might not be feasible in another. Examples of differences occurred in the management of instrumentation bias (S1 Table) or in blinding strategies used (Table 2). As a community, we termed this study design information the “workflow.” The workflow data are recorded annually via a survey using the MEDO ontology. The practical reality of experimental design is that there is no perfect solution; therefore, transparency is needed to capture how experiments were implemented to allow users to independently assess the potential strengths and weaknesses in the design. This level of transparency also encourages good practice within the consortium. For example, the Japan Mouse Clinic switched from an “alternate mouse” method to a “cage active randomisation method” to minimise potential order effects when assaying mice.

**Table 1:** The various control design options available and associated definitions.

Control design option	Definition
**Line mate control**	The process of using control WT mice that are generated from a breeding programme of HET*HET or HET*WT crosses used to generate the mutant of interest.
**Littermate control**	The process of using WT mice that are true siblings and hence were generated from a HET*HET, or a HET*WT cross that generated the mutant of interest. If HET mice are the controls, the control mice are true siblings from a HOM*HET or a HET*HET cross that generated the mutant of interest.
**Pooled genetic control**	The process of using WT mice from HET*HET or HET*WT breeding that generates mutant mice. This can be from matings that generate a variety of KO alleles, all of which are on the same genetic background.
**Production colony control**	A process in which WT mice from a breeding colony of the same genetic background are used.

The table lists the various ontologies and associated definitions that were developed to describe the control strategies used in the implementation of the phenotyping experiments. WT indicates wild type, KO indicates knockout, HOM indicates homozygous, and HET indicates heterozygous.

**Table 2:** Potential blinding strategies and associated definitions used to describe the experiments.

Blinding strategy	Definition
**Blinded**	A process in which the person performing the phenotyping does not have access to either genotype or allele information.
**Unblinded**	A process in which the experiment is run with no blinding, i.e., both genotype and allele are visible to the person performing the phenotyping procedure.
**Genotype free blinding**	A process in which the person performing the procedure has access to the allele, but not the genotype information.
**Allele free blinding**	A process in which the person performing the procedure knows the genotype information but not the allele information of the subject being phenotyped

The table lists the various ontologies and associated definitions that were developed to describe the blinding strategies implemented in phenotyping experiments.

The standardised language developed is specific to the variation in implementation that could occur within our community, but also to variation sources that were felt to have the potential to have a biological impact on the experimental outcome that were not controlled by standardisation within our studies. As a result, the language we developed is not generic but could form a starting point for other communities.

### ARRIVE Guidelines—Methods—Sample Size (Item 10)

As a high-throughput project, the target sample size of 14 animals (seven per sex) per knockout strain is relatively low. This number was arrived at after a community-wide debate to find the lowest sample size that would consume the least amount of resources while achieving the goal of detecting phenotypic abnormalities. At times, viability issues or the difficulty in administering a test might further limit the number of animals. As such, whenever data are visualised, the number of animals phenotyped is listed.

In a high-throughput environment, replication of individual lines is not cost effective. Instead, we are generating and characterising a common set of six “reference” knockout lines that will present a wide range of phenotypes based on previously published research.

### ARRIVE Guidelines—Methods—Statistical Methods (Item 13)

Ensuring that the appropriate statistical analysis is applied is a common problem in biology [9]. For high-throughput phenotyping platforms, this is an area of active research [10,11]. Developing an analysis pipeline for a resource with data from many sources is challenged by the number of variables, different data types, the data quantity for an institute, and variation in experimental workflow. One example of variation in workflow is in the difference between data from the Institut Clinique de la Souris, which is collected with a design of one batch of knockout mice with concurrent controls, and that of the Wellcome Trust Sanger Institute, in which the knockout and control mice are collected in multiple batches and not necessarily on the same day. The analysis is further complicated by the requirement that it be completely unsupervised, with no user intervention. As such, an analysis pipeline has to be robust, it must process the data consistently, and it cannot be fine-tuned for all possible scenarios.

To address the analysis questions, the international community has come together to form an IMPC Statistical Technical Group to systematically address these issues and inform the research that is needed in this area. Consequently, we have implemented an analysis pipeline that applies the best statistical tests based on the assay and the structure of the data (e.g., control method used). To ensure this analysis is transparent, we have developed a package of tools called PhenStat that uses the popular statistical language R. The package is freely available from Bioconductor (http://www.bioconductor.org/packages/release/bioc/html/PhenStat.html), and the application is version-controlled, as there is still active research ongoing in this area of data analysis. We also provide an explanation of the analysis, which can be accessed from the IMPC ARRIVE landing page.

On the web portal, each graphical representation includes a statistical summary with *p*-values and effect sizes, with the ability for users to obtain more information about the statistical method implemented (S1 Fig). As data can be analysed in many ways, and each way has strengths and weaknesses, we have developed tools and procedures to enable users to obtain raw data for independent analysis, either via spreadsheet downloads, file transfer protocol (ftp) access, or automatic programmatic interfaces.

### ARRIVE Guidelines—Results—Numbers, Baseline Data, and Outcomes and Estimation (Items 14–16)

The challenge with the analysis is not only in selecting the most appropriate analysis platform but in presenting all the results and the concomitant information in a user-accessible way. Accompanying the graphical descriptions of the data are summary measures for each group of mice.

Data are rarely excluded from the analysis. Exclusion can arise from two mechanisms. Firstly, data points may fail quality control (QC) during data collection. An explanation is provided using a standardised set of options as agreed by area experts. An example is the explanation “Procedure Failed—Insufficient Sample—Blood sample.” Secondly, data are QC’d after upload from the institute to the database, where data of concern are investigated using Phenodcc, a set of tools that visualise the data and track concerns. This approach is essential when dealing which large volumes of data. Data are only QC-failed if clear technical reasons can be found for a measurement being an outlier. Reasons for QC failure are provided and tracked within the database.

### ARRIVE Guidelines—Discussion—Interpretation / Scientific Implications (Item 18)

Results are interpreted automatically following statistical analysis by the assignment of a Mammalian Phenotype Ontology term (MP term) when a significance threshold (*p* < 0.0001), agreed upon by the community, is reached. The Mammalian Phenotype Ontology is a standardised ontology developed by the Mouse Genome Informatics group for describing a phenotype. The use of the standardised ontology is critical to allow comparison across studies and species.

The ARRIVE guidelines invite comments on the study limitations, including any potential sources of bias and imprecision (Item 18b). This is an area of active discussion amongst the IMPC Statistical Technical Group. For example, the group is considering methodologies to estimate the false discovery rate.

### ARRIVE Guidelines—Discussion—Generalizability / Translation (Item 19)

Mouse repositories play an important role in the generalizability of results by allowing researchers to perform follow-up studies on genetically identical animals to those used in previous analysis. All strains generated by the IMPC are available to the research community via the established mouse repositories (e.g., Knockout Mouse Repository). These repositories include details about allele structure, genetic background, pathogen exclusion list and any potential issues in husbandry and welfare that may result from carried mutations. This information increases the likelihood of reproducing previous results, which is important for translating mouse research to humans [5].

Many animal experiments study only one sex, typically the males, to avoid potential issues with oestrogen cycles. There has been growing concern over the sex imbalance in biomedical research [12–14] and this had led to the United States National Institutes of Health now requiring all grant applicants to consider both sexes in preclinical studies [15]. Fortunately, IMPC decided at the start of the project that phenotype data should be collected from both sexes, and this design supports the generalizability of any findings. In addition, our continuous analysis pipeline is designed to detect sexual dimorphism and we associated a tag to the MP terms to classify the effect observed (S2 Fig).

Mouse knockout (or null) mutations lead to the absence of the gene product and are recognized as a standard approach in all model organisms for assessing gene function. However, not all aspects of gene function will be revealed by the generation of a knockout mutation. For example, gain of function effects that might arise from the introduction of a specific amino acid change in a protein would not be uncovered. Nevertheless, many Mendelian disorders in the human population, including rare diseases, will be caused by severe loss of function effects for which the data generated from mouse knockout mutations will be highly informative. Moreover, recent studies [16] have found a large number of associations between the phenotypes of Mendelian and complex diseases that link complex disorders to a unique spectrum or “code” of Mendelian loci. In addition, common variants associated with complex disease are enriched in those Mendelian loci. This underscores the wider utility of examining loss-of-function alleles in the mouse. As a translational resource, we have developed an automated analysis pipeline to detect phenotypic similarities between inherited human diseases and our knockout mouse lines. This pipeline uses a semantic matching algorithm termed PhenoDigm to detect and score similarities between human clinical observations and mouse phenotypes [17]. We present the links between human disease and IMPC lines on our website, along with the underlying evidence. Lines with interesting disease relationships can then be ordered for further research into the pathobiology of the disease and drug target validation.

## Conclusions

Applying the ARRIVE guidelines for a phenotype resource generated by an international community introduced many challenges, some specific to a data resource and others that are more general.

The first set of challenges arose in applying guidelines intended for a scientific publication to a data resource. The detail needed to meet the guidelines required significant organisation and informatics infrastructure to enable the capture, QC, analysis, and storage of the raw and derived information. To do this, the IMPC developed several resources: the IMPReSS database, which standardizes procedures and captures information needed to reproduce experiments; the PhenStat analysis package, which defines the statistical procedures to enable others to replicate analysis; iMITS, which tracks mouse production and phenotyping milestones; and finally, Phenodcc, which supports QC of the data. We have also developed tools to support data download to allow data to be analysed independently. Another issue specific to data resources is the presentation of the large volume of data necessary to be transparent. Through a user-driven design approach, we developed a web portal that gives an overview of gene-phenotype associations with the ability to drill down to details that include graphs, statistical analysis results, and numbers of animals involved. We have also constructed an ARRIVE page (https://www.mousephenotype.org/about-impc/arrive-guidelines) that consolidates all the information. These efforts have provided the transparency that is essential to allow reflective assessment of our data.

A second, more general set of issues reflects a lack of standards in how experiments are described. This necessitated the development of a standardised language with defined terms and definitions, coupled with the use of surveys to clarify each centres’ implementation strategy. Our experiences are likely to reflect a general issue in the biological community and could help explain the lack of progress in applying the ARRIVE guidelines [9]. As a first step, we needed to give priority to detailed explanation of experimental implementation. The development of a standardised language has allowed us to capture and provide transparency in the experimental design, which also encourages best practice.

The ARRIVE guidelines have provided a framework to consider our ethical responsibilities in assessing what is necessary to communicate to the consumers of our data. Meeting the guidelines involved the development of multiple databases, bespoke software packages, and an ontology. Applying the ARRIVE guidelines has proven to be a challenge, but the resulting transparency has encouraged good practice and allows IMPC phenotype data to be fairly evaluated. The effort has been worthwhile and the value, both internally and externally, is clear. However, it also highlights the scale of effort needed for the general biological community to improve reporting of in vivo experiments.

## Materials and Methods

For the example presented in S1 Fig, looking at the impact of the *Nptn* gene knockout on the lean mass phenotype, mice carrying the *Nptn* ^*tm1b(EUCOMM)Hmgu*^ targeted allele were created by blastocyst injection of targeted ES cells, and bred on the C57BL/6NTac genetic background (accession number: MGI:5548382). Phenotyping data was collected on nine female and eight male homozygous mice and 1,079 female and 1,107 male baseline control mice collected during the project.

### Ethics Statement

The care and use of all mice in the *Nptn* study were carried out in accordance with United Kingdom Home Office regulations and the Animals (Scientific Procedures) Act of 1986 Amendment Regulations 2012 (SI 4 2012/3039). This study has been approved by the Animal Welfare and Ethical Review Board (AWERB), resulting in the licence 30/2890. All efforts were made to minimize suffering by considerate housing and husbandry, the details of which are available at the IMPC portal: http://www.mousephenotype.org/about-impc/arrive-guidelines.

Animal welfare was assessed routinely for all mice involved. Adult mice were killed by terminal anaesthesia followed by exsanguination, and death was confirmed by either cervical dislocation or cessation of circulation.

### Dual-Energy X-ray Absorptiometry (DEXA)

At 14 weeks of age, the DEXA procedure was followed as detailed in the standardised protocol specified at https://www.mousephenotype.org/impress/protocol/90/7 using a Lunar Piximus II Bone Densitometer (GE Medical Systems, UK) after the animals were anaesthetized with isoflurane (2% Abbott Animal Health, US). The details of blinding and randomisation methods implemented are described at http://www.mousephenotype.org/about-impc/arrive-guidelines.

## Supporting Information

S1 Fig Organising the information for rapid user access. Organising the information in a web user interface is challenging. Shown are screen shots from a genotype-phenotype page to highlight how the information is organised and presented. A: An example visualisation of phenotyping data. B: An example presentation of associated statistical output. The example shown is the lean mass output from the Dual Energy X-ray Absorptiometry screen for the *Nptn* ^*tm1b(EUCOMM)Hmgu*^ knockout line (accession number: MGI:5548382).
(TIFF)

S2 Fig Classification of the observed genotype effect. As the majority of the phenotyping data are collected on both sexes, this has enabled the regression methods to include an assessment of sexual dimorphism. The output of the model fitting can then be used to classify the genotype effect observed in relationship to the sex of the animals. For example, whether the genotype effect was observed in both sexes equally will lead to the tag “both sexes equally” or specifically to one sex (e.g., “males only”). Occasionally the model optimisation procedure implemented will find that there was statistical evidence of sexual dimorphism but when it came to identifying how this occurred and quantifying the effect for each sex, there is insufficient power. In this scenario, the classification returned states that it “cannot classify the effect.”
(PDF)

S1 Table The various instrument bias management options available to describe how institutes manage potential instrument effects. The table lists the various ontologies and associated definitions that were developed to describe the strategies used in the implementation of the phenotyping experiments to manage the potential effect of instrument bias.
(PDF)

## Abbreviations

ARRIVE: Animal Research, Reporting of In Vivo Experiments
GA: Genetically Altered
HET: heterozygous
HOM: homozygous
iMITS: International MicroInjection Tracking System
IMPC: International Mouse Phenotyping Consortium
IMPReSS: International Mouse Phenotyping Resource of Standardised Screens
KO: knockout
MEDO: Mouse Experimental Design Ontology
MP term: Mammalian Phenotype Ontology term
QC: Quality control
WT: wild type
