# Supplementary figures and images for: Applying the ARRIVE Guidelines to an In Vivo Database
Source: PLoS Biol. 2015 May 20;13(5):e1002151. doi: 10.1371/journal.pbio.1002151 (PMC4439173; doi:10.1371/journal.pbio.1002151)

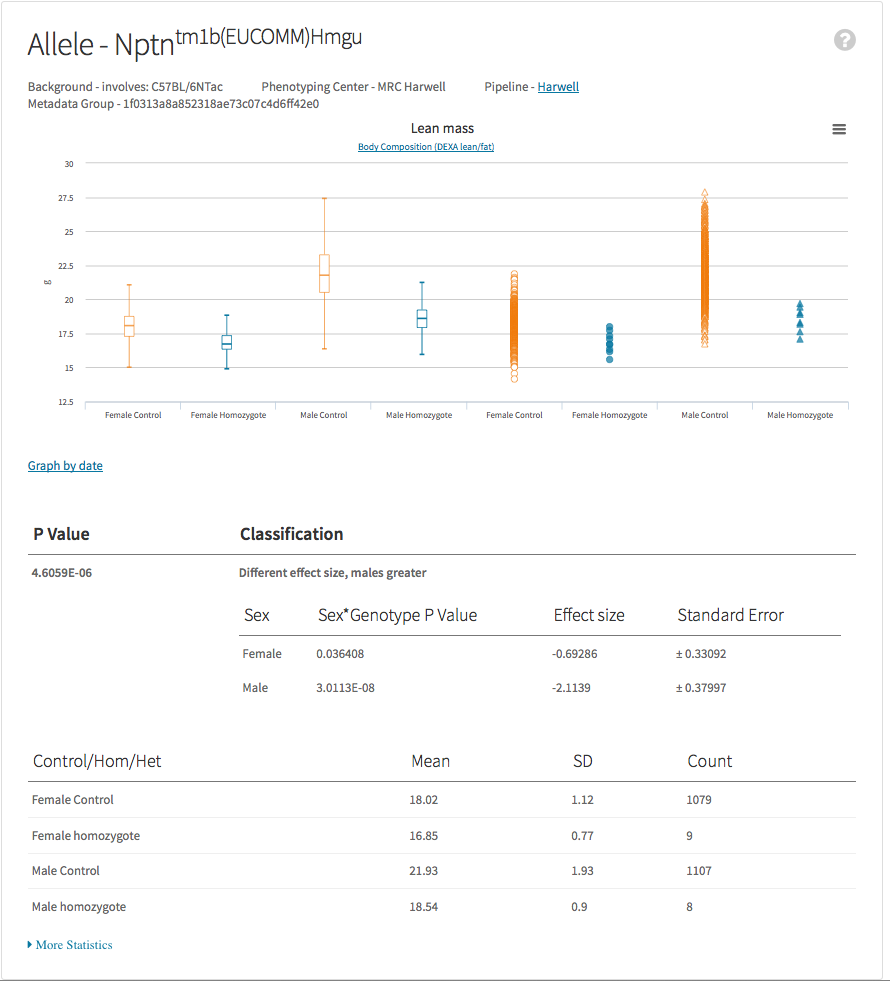

Supplement: S1 Fig — Organising the information in a web user interface is challenging. Shown are screen shots from a genotype-phenotype page to highlight how the information is organised and presented. A: An example visualisation of phenotyping data. B: An example presentation of associated statistical output. The example shown is the lean mass output from the Dual Energy X-ray Absorptiometry screen for the Nptn tm1b(EUCOMM)Hmgu knockout line (accession number: MGI:5548382). (TIFF) [file pbio.1002151.s001.tiff]

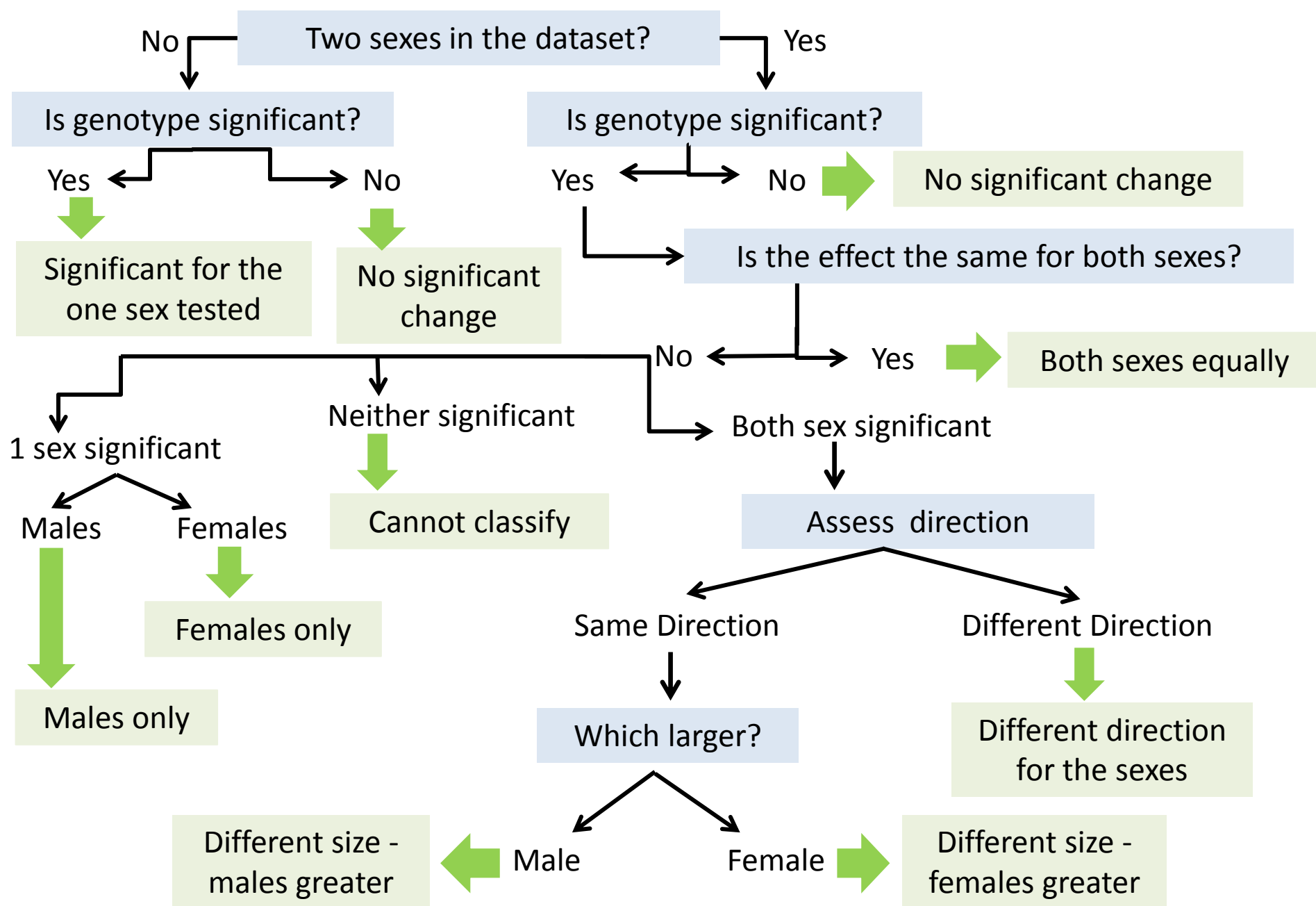

Supplement: S2 Fig — As the majority of the phenotyping data are collected on both sexes, this has enabled the regression methods to include an assessment of sexual dimorphism. The output of the model fitting can then be used to classify the genotype effect observed in relationship to the sex of the animals. For example, whether the genotype effect was observed in both sexes equally will lead to the tag “both sexes equally” or specifically to one sex (e.g., “males only”). Occasionally the model optimisation procedure implemented will find that there was statistical evidence of sexual dimorphism but when it came to identifying how this occurred and quantifying the effect for each sex, there is insufficient power. In this scenario, the classification returned states that it “cannot classify the effect.” (PDF) [file pbio.1002151.s002.pdf]
