# Supplementary material for: Applying the ARRIVE Guidelines to an In Vivo Database
Source: PLoS Biol. 2015 May 20;13(5):e1002151. doi: 10.1371/journal.pbio.1002151 (PMC4439173; doi:10.1371/journal.pbio.1002151)
Supplement: S1 Table — The table lists the various ontologies and associated definitions that were developed to describe the strategies used in the implementation of the phenotyping experiments to manage the potential effect of instrument bias. (PDF) [file pbio.1002151.s003.pdf]

**Table S1: The various instrument bias management options available to describe how institutes manage potential instrument effects.**

The table lists the various ontologies and associated definitions that were developed to describe the strategies used in the implementation of the phenotyping experiments to manage the potential effect of instrument bias.

| Instrument bias management | Definition |
|---|---|
| **active randomisation and minimisation instrumentation strategy** | The process by which instrument differences are managed by randomly assigning the subjects, processed within a defined time frame of one day, to the different instruments using a randomisation technique (e.g. alternate allocation or odd or even last digit of sample number). Steps are also taken to minimise the potential effect of differences in instrumentation e.g. calibration of scales between instruments. |
| **active randomisation instrumentation strategy** | The process by which instrumentation differences are actively managed by randomly assigning subjects (e.g. mice), processing these in a defined time frame of 24 hours, with assignment managed by a randomisation technique such as alternate allocation, or odd/even last digit sample number allocation. |
| **balanced and minimisation instrumentation strategy** | The process by which instrument effects are managed. Instrument effects will affect subjects (e.g. mice) equally as equal numbers of the control and knockout mice are processed by a specific instrument. Steps are also taken to minimise the potential effect of differences in instrumentation e.g. calibration of scales. |
| **balanced instrumentation strategy** | The process by which instrument effects affecting the ‘local’ subjects are managed. These affect the local subjects (e.g. control and knockout mice) equally as equal numbers of subjects of different genotypes are processed by a specific instrument. |
| **casual randomisation and minimisation instrumentation strategy** | The process by which instrument differences are managed by randomly assigning subjects, processed within a defined time frame of 24h, to the different instruments using an operator to randomly place the mice. Steps are also taken to minimise the potential effect of differences in instrumentation e.g. calibration of scales. |
| **casual randomisation instrumentation strategy** | The process by which instrument differences are managed by randomly assigning the subjects, processed within a defined time frame of 24h, to the different instruments using an operator to randomly place the mice. |
| **controlled instrumentation strategy** | The process by which instrumentation effects are minimised as the same instrument is used for all subjects. |
| **minimisation instrumentation strategy** | The process by which steps are taken to minimise the potential effect of differences in instrumentation e.g. calibration of scales. |
